# Systematic Review of Efficacy of Interventions for Social Isolation of Older Adults

**DOI:** 10.3389/fpsyg.2021.554145

**Published:** 2021-09-07

**Authors:** Feng Tong, ChengLin Yu, LinSen Wang, Iris Chi, Fang Fu

**Affiliations:** ^1^School of International Law and Sociology, Sichuan International Studies University, Chongqing, China; ^2^School of Humanities and Management, Southwest Medical University, Sichuan, China; ^3^Suzanne Dworak-Peck School of Social Work, University of Southern California, Los Angeles, CA, United States; ^4^School of Social Development and Public Policy, Fudan University, Shanghai, China

**Keywords:** social isolation, loneliness, older adults, health, systematic review, intervention

## Abstract

**Background:** The social isolation of older people is a growing public health concern. The proportion of older people in society has increased in recent decades, and it is estimated that ~40% of the population will be aged 50 or above within the next few decades. This systematic review aims to summarize and renew knowledge of the effectiveness of existing interventions for alleviating social isolation of older adults.

**Methods:** Relevant electronic databases, including Cochrane Library, CINAHL, SCOPUS, and Web of Science, were searched by a systematic evaluation method. Eligible randomized controlled trial (RCT) studies were published between 1978 and 2021 in English or Chinese. The primary and secondary outcomes were social isolation and loneliness. The quality of the included RCTs was scored by the Cochrane risk-of-bias tool to assess their quality. Two independent reviewers extracted data, using a standardized form. Narrative synthesis and vote-counting methods were used to summarize and interpret study data.

**Results:** Twenty-four RCTs were finally included in this review. There was evidence of substantial heterogeneity in the interventions delivered. The overall quality of included studies indicated a low-to-medium risk of bias. Eighteen of 24 RCTs showed at least one dimension effect on reducing social isolation. The interventions with accurate targeting of clients in social and public places had more obvious effect. The interventions in which older people are active participants also appeared more likely to be effective. In addition, group intervention activities and individual intervention interviews were effective in improving structural social support; mixed intervention, and group intervention on training support significantly improved functional social support.

**Conclusions:** This study suggests that group and mixed intervention targeting of older adults could be helpful for alleviating social isolation problems. The use of modern technology for remote services could also present good results. More well-conducted RCTs of the effectiveness of social interventions for alleviating social isolation are needed to improve the evidence base. Especially as the debating results of remote interventions, further research in this field should be conducted.

## Background

Social isolation is a major threat to the health of older adults. There are many risk factors in social isolation in old age, including the lack of family members, rare or no daily communication with friends, depression, and a solitary lifestyle (Iliffe et al., 2007). Studies have indicated that social isolation and loneliness are common negative emotions, and social states among older adults that could lead, without timely intervention, to even more serious situations (Laursen and Hartl, 2013). Subsequently, research has indicated that 40% of adults over the age of 50 often felt lonely (Ferreiraalves et al., 2014). Although “loneliness” is often co-emergent and mutually influential with “social isolation,” they are two different concepts (Grenade and Boldy, 2008). Loneliness relates specifically to negative feelings of one about a situation. It may reflect social isolation or a sense of abandonment, resulting from an excessive gap between expectations and reality (Petersen et al., 2020) and increase with age (Li and Zhou, 2002), while the definitions of social isolation incorporate “structural” and “functional” social support (Lu et al., 2013). Social isolation is, therefore, multidimensional and includes the lack of structural and functional social support (Lubben and Gironda, 2003; Victor et al., 2010). In this research, social isolation was divided into two dimensions: “structural social support” and “functional social support.” Structural social support is an objective evaluation of the scale or frequency of social support participation (Lubben and Gironda, 2003; Victor et al., 2010); and functional social support is a subjective judgment on the quality of social support, including feelings, tools, and information provided by the perceived responses of others (Hall et al., 2019). According to this definition, social isolation is a multidimensional concept, which results from the lack of quality and quantity of social support (Petersen et al., 2020). The current study adopted this definition as the basis for research.

Social isolation is an essential threat to the health of older adults, and many scholars have provided evidence for methods of alleviating this problem. A meta-analysis conducted in 2010 (*n* = 308, mean age = 64 years; Lunstad et al., 2010) indicated that social isolation of people with strong social relationships might decrease by 50%. The compound variables used to calculate “strong social relationships” included loneliness and social isolation. Specific studies assessing the relationship between social isolation and health have reached different conclusions. For example, social isolation can lead to increased mortality, worse self-rated health (Cornwell and Waite, 2009), more susceptibility to Alzheimer's disease (Fratiglioni et al., 2000), and an increased rate of disability in older adults (Lund et al., 2010). A recent study suggested that social isolation was negatively correlated with health-related quality of life and health status of older adults (Hawton et al., 2010). Much evidence has accumulated to indicate that social isolation can affect the health of an individual. Therefore, it is an important public health problem. Moreover, the results of interventions for social isolation must be scientifically evaluated to reduce its negative impact.

There are several systematic reviews of this topic. For example, one study summarized interventions for loneliness. However, it does not fully address the effectiveness of interventions for social isolation (Masi et al., 2011). In this article, data were integrated from heterogeneous samples and included out-of-school children, homeless teenagers, and older adults. Moreover, the interventions included online chat rooms, exercise, social events, and training support. Although there is a debate about the appropriateness of meta-analysis of heterogeneous data, this kind of systematic evaluation of outcome research has seldom been reported. Recently, two systematic reviews have been conducted that included studies before 2016 (Stojanovic et al., 2016; Poscia et al., 2018). However, in these two systematic reviews, there was no quality evaluation process, and RCTs were not included. Moreover, they did not search the three main databases of PsycINFO, PubMed, and Proquest. Since then, many changes have taken place in the social environment. Remote services have been widely adopted, especially with the rapid development of information technology. Remote and other new-tech intervention RCTs targeting social isolation in older adults have been published until 2021, which necessitates updating of current knowledge.

Outcomes regarding structural social support and functional social support are important indicators of effect in the multidimensional definition of social isolation used in our review. In addition, reporting on loneliness may also contribute to the understanding of intervention effects. Therefore, this systematic review was designed to summarize and update the current knowledge about the efficacy of existing interventions for alleviating social isolation and loneliness among older adults.

## Methods

### Search Strategy

The literature published from January 1978 to January 2021 was systematically retrieved, using ENDNOTE X6, to manage the literature. Electronic database retrieval included PsycINFO, PubMed, Proquest, Cochrane Library, Applied Social Sciences Index and Abstracts (ASSIA), CINAHL databases, SCOPUS, Web of science, China National Knowledge Infrastructure (CNKI), and Wanfang Data Knowledge Service Platform (WANGFANG). Another search retrieved social isolation and/or loneliness in the review and has been included in the study of the reference literature; retrieval from the University of Southern California Social Work Institute database, evidence-based medicine research center of Lanzhou University, and Population Research Institute of Southwestern University of Finance and Economics. Contacts were made with scholars within the network of the authors to obtain information about ongoing studies. Search words used were as follows: older/elder/senior/aged/geriatric, isolation/isolate/isolated, lone/loneliness/alone/solitude/solitary, social support/network/relations, psychosocial intervention, treatment/therapy, clinical trial, explanatory trial, pragmatic trial, and randomized controlled trial. Search terms used were as follows: (isolation/isolate/isolated) or (lone/loneliness/alone/solitude/solitary) or (social support/network/relations) and (older/elder/senior/aged/geriatric) and (intervention/therapy/clinical trial/explanatory trial) or (explanatory trial/pragmatic trial/randomized controlled trial). The search terms in different databases were slightly different. Therefore, we also searched through the reference lists of systematic review articles on social isolation.

### Review Strategy

According to the research topic and summary, two researchers (FT and CLY) made a preliminary identification of study criteria. The third researcher (FF) read the abstract of the indeterminate literature and determined the specific discussion about the disagreement. A pair of independent raters selected abstracts for full review based on inclusion/exclusion criteria. Two independent reviewers extracted data, using a standardized form. Due to the heterogeneity of different outcome indicators (e.g., family ties increased, feeling of social support, social relationship), meta-analysis is not suitable for use. According to the analysis method of the three previous evaluations (Díaz and García, 2016; Canedo-García et al., 2017; Li et al., 2018), narrative synthesis and vote-counting methods were used to summarize and interpret study data. The current review was reported in accordance with the latest PRISMA guidance (Page et al., 2021).

### Inclusion and Exclusion Criteria

The primary and secondary outcomes are social isolation and loneliness. All papers selected for final inclusion met the following criteria: (i) older adults over 50 years of age with no mental illness or cognitive impairment; (ii) the purpose of the intervention was to alleviate social isolation or loneliness; (iii) the results of social isolation intervention were reported; (iv) there were randomized controlled trials but no drug trials; and (v) the paper was written in Chinese or English. Exclusion criteria for the study: (i) study samples aged younger than 50 years; (ii) not used a randomized controlled trial (RCT); (iii) drug intervention was used; and (iv) outcomes reporting only on loneliness but no social isolation.

### The Quality Evaluation of the Research

Because of the heterogeneity of the intervention types and results of the trial, quantitative analysis of data was not used in the review, so the method of narrative synthesis was applied to analyze the effect of interventions. In the quality evaluation of open randomized controlled trials, we chose not to use the Jadad standard (Berger, 2006) as this is focused on blind and random sequences; therefore, the Cochrane risk-of-bias tool was deemed more appropriate (Ma et al., 2012). In this paper, based on the Cochrane risk of bias, the quality of the randomized control trial and the bias risk level were identified, and the grading principle of JADAD was used to evaluate the overall research quality. The Cochrane bias-risk tool evaluation principle involves six aspects: selection bias, implementation bias, measurement bias, data bias, publication bias, and other bias (Higgins et al., 2011). The system evaluation report is based on the PRISMA (preferred reporting items for systematic reviews and meta-analyses) standard (Page et al., 2021).

## Results

**Figure 1:**
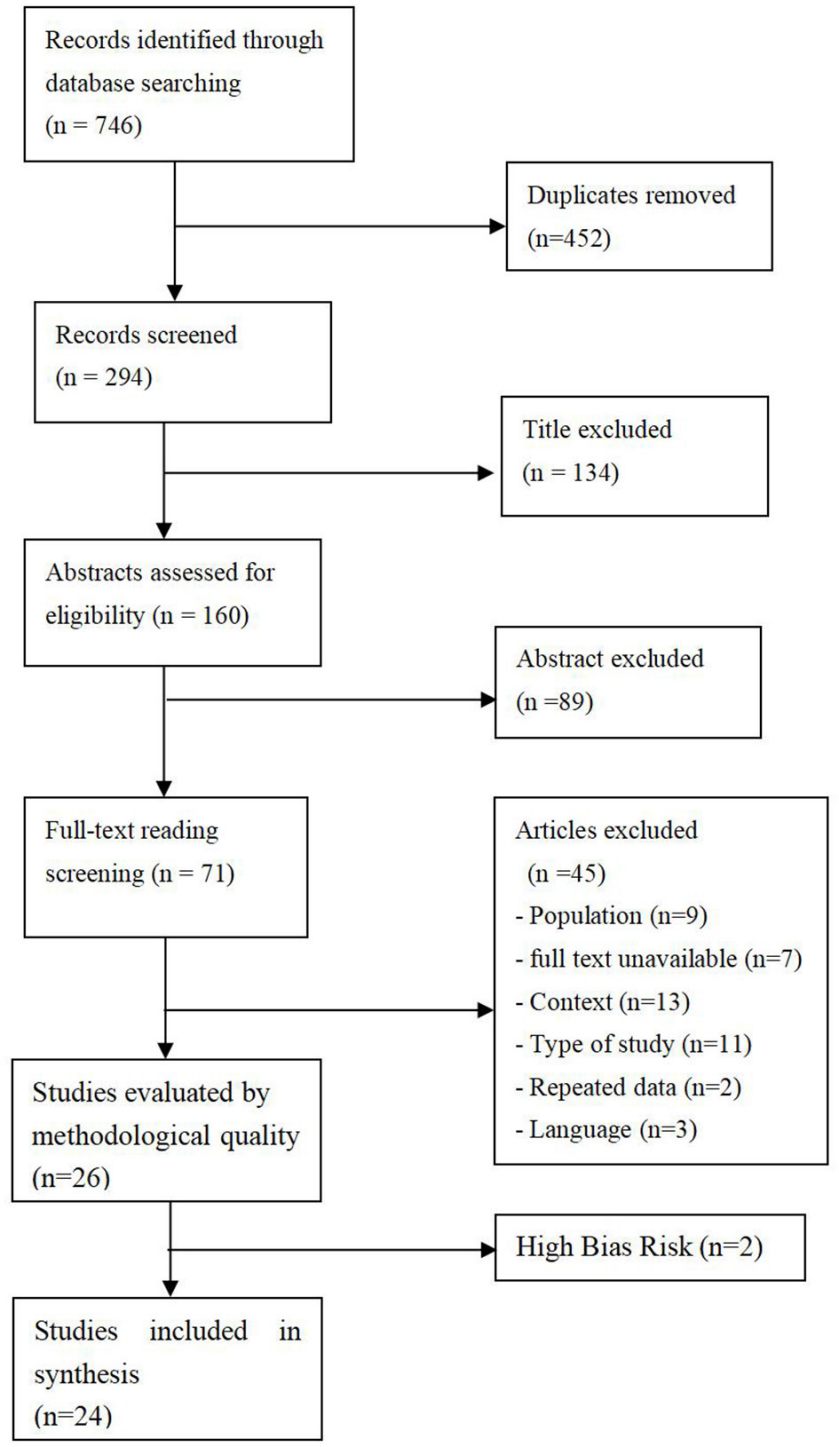
Eligibility and screening of studies considered for the systematic review.

About 746 items were found in the related research, with 452 duplicates removed, 268 of the studies excluded as they did not meet the selection criteria. Two studies were excluded because of high-bias risk. Twenty-four studies were eventually included (Figure 1).

### Inclusion of the Study

A total of 24 randomized controlled trial studies were included with a total of 4,078 subjects, each involving 26–708 cases. Table 1 (including two high-bias risks) introduces the characteristics of these studies in accordance with the principle of PICOS, including: population, intervention, comparison, outcomes, and study type (Methley et al., 2014).

**Table 1:** Characteristics of studies stratified by PICOS.

**Authors, time (country)**	**Population**	**Intervention**	**Comparison**	**Outcome**	**Study type**
Harris and Bodden (1978) (USA)	102 cases of incapacitating elderly in the community The mean age is 77	Group intervention activities	Conventional intervention	Chicago Social Activity Scale	Community/public places
Constantino (1988) (USA)	150 widowed women I1 (50)/I2 (50)/C (50) The mean age is 58	Group intervention activities	Not described	RSAS,E(DACL)	School/established place
Lökk (1990) (Sweden)	65 cases of community living disabled elderly (33/C (32) Average age I (76)/C (78) Female ratio I (52%)/C (50%)	Group intervention activities	Standard recovery project	Outdoor activity index, social network index	Care center
Ollonqvist et al. (2008) (Finland)	708 cases of community elderly I (343)/C (365) Average age 78 Female ratio I (85%)/C (87%)	Group intervention activities	No intervention	Loneliness, satisfaction with contact with children, the number of friends and relatives	Rehabilitation center
Routasalo et al. (2009) (Finland)	235 cases of community living in the elderly with lonely orientation I (117)/C (118) Average age 80 Female ratio I (74%)/C (73%)	Group intervention activities	No intervention	UCLA, Lubben's social networking scale, and social activity and mental health status	Community center/public place
Black et al. (2014) (USA)	26 Tai chi elderly people scored ≥40 loneliness scale	Group intervention activities	Not described	psychological Stress Scale, social network index	Community
Chan et al. (2017) (HongKong)	48 elderly persons, not engaged in any social activities Mean age: 77.3 years Women:76%	Group intervention activities	Regular home visits by social workers.	The Lubben social network, De Jong Gieveld loneliness scales, social support questionnaire	Home, community
Ristolainen et al. (2020) (Finland)	345 elderly persons, age 65+ I(159)/C(186); famle radio I (82.2%)/C(83.6%)	Group intervention activities	No intervention	UCLA DOM3	Not clear
Fukui et al. (2003) (Japan)	50 cases of early breast cancer women I (25)/C (25) Average age is 53	Group intervention training support	Candidate interventions	UCLA, questionnaire	Hospital
Savelkoul and de Witte (2004) (Norway)	168 cases of chronic rheumatic patients with I (56)/C1 (56)/C2 (56) Average age 53/52/51 Female ratio I (77%)/C1 (59%)/C2 (73%)	Group intervention training support	Mutual help group	de Jong Gierveld, social support interactive table	Not clear
Kremers et al. (2006) (Norway)	142 Community Single Women I (63)/C (79) Average age I (63)/C (66)	Group intervention training support	No intervention	de Jong Gierveld	Not clear
Bøen et al. (2012) (Norway)	138 patients from 14 elderly centers (77)/C (61). Intervention group 80 years old accounted for 60%, controlled group 80 + accounted for 50% Female ratio I (60%)/C (55%)	Group intervention training support	No intervention/daily activities	SF-36, CES-D, HSCL-10	Geriatric center
Saito et al. (2012) (Japan)	63 elderly people who have settled for more than 2 years after migration (42)/C (21) Mean age I (73)/C (73) Female ratio I (60%)/C (70%)	Group intervention training support	Waiting list	LSI-A,GDS, AOK, and loneliness and social support	Established public place
White et al. (2002) (USA)	100 nursing homes and concentrated elderly people I (51)/C (49) Average age I (71)/C (72) Female proportion I (71%)/C (82%)	Groups intervene in remote services	Conventional care	UCLA, Number of close friends, CES	In nursing homes/housing
Schulz (1976) (USA)	Residents I1 (10)/I2 (10)/C1 (10)/C2(10) of 40 churches Average age I1 (85)/I2 (80)/C1 (83)/C2 (78) Women 90%	Individual intervention interview	(1) random access (2) no intervention	The activity index, Wohlford hope scale	Geracomium
MacIntyre et al. (1999) (Canada)	26 home care or home care beneficiaries were I (15)/C (11) The mean age was I (80) and/C (79) The female ratio was I (58%)/C (80%)	Individual intervention interview	Routine intervention	Personal resource questionnaire	Private residence
Yi et al. (2012) (China)	144 cases of community aged and elderly living in the home were I (74)/C (70) The mean age was I (85) and /C (84) The female ratio was I (80%)/C (76%)	Individual intervention interview	Door-to-door service according to demand.	GDS-15, UCLA	Private residence
Heller et al. (1991) (USA)	565 cases of low-income families female I (291)/C (274)	Individual intervention in remote service	No intervention	Paloutzian and ellison loneliness scale, CES-D, SSRS	Private residence
Brennan et al. (1995) (USA)	102 patients with community-owned Alzheimer's disease (AD) patients I (51)/C (51) Average age is 64 Female Proportion is 67%	Individual intervention in remote service	Local community service	CES-D, Instruments and emotional social support scale, social contacts, and records of medical services	Private residence
Morrow et al. (1998) (USA)	61 elderly patients with suicidal tendencies were I (30) and/C (31) The average age was 76 The female ratio was 85%	Individual intervention in remote service	Waiting list	GDS, OARS, Social isolation	Private residence
Slegers et al. (2008) (Norway)	107 cases without Internet experience community elderly I (62), /C1 (45)/C2 (68), /C3 (61)	Individual intervention in remote service	Remote service not accepted	de Jong Gierveld, SF-36, The symptom checklist	Private residence
Mountain et al. (2014) (UK)	70 subjects, with good cognitive function, lived independently in a British city. Women's ratio I (66%)/ C (51%)	Mixed intervention in remote service	Not described	SF-36,MH	Home
Czaja et al. (2017) (USA)	300 elderly people at risk of social segregation, Average age I (76.9)/C (75.3) Proportion of women I (79.3%)/C (76.7%)	Mixed intervention in remote service	No intervention	Social isolation Index, Social support Index, computer proficiency Index and attitude toward Technology	Personal residence
Drentea et al. (2006) (USA)	Of 183 patients with Alzheimer disease (AD), I (94)/C (89) The mean age was I (73) and/C (71) The female ratio was I (58%)/C (66%)	Mixed intervention	conventional therapy	SSNL, Social support satisfaction	Private residence
Hang et al. (2011) (China)	There were 80 empty nest elderly patients with depressive symptoms, I (40), and/C (40) The mean age was I (72) and/C (71) The female ratio was I (58%)/C (50%)	Mixed intervention	No intervention	GDS, UCLA, MUNSH	Community (Group)/private residence (individual)
Lai et al. (2020) (Canda)	60 community-dwelling older Chinese immigrants aged 65 and older I (30)/C (30). The female ratio I(66.7%)/C(60%)	Mixed intervention	Received brief telephone calls	DJLS-6, LSNS	Not clear (Group)/private residence (individual)

*I (N), intervention group (N), C (N), control group (N); N, number of objects or proportion; subjects were the number of baseline or pretest interventions. RSAS, the social adaptation scale; BDI, Duke depression scale; (DACL), E; depression adjective checklist; GDS, geriatric depression scale; SF, short form health survey; CES-D, CES-D; HSCL psychological symptom checklist, self-rating scale; DJLS-6, De Jong loneliness scale-6; OARS, Duke University table SSNL; social network scale; LSI-A, self-rating scale, life satisfaction index; AOK loneliness scale; MUNSH, Memorial University of Newfoundland scale of happiness*.

Of all the studies, there were only two studies from mainland China (Hang et al., 2011; Yi et al., 2012), and one of them belonged to high risk of bias. The rest of the studies were from Hong Kong, Europe, and other developed countries. The United States occupied 10 studies, while the low-risk bias research was mostly from Finland (Ollonqvist et al., 2008; Routasalo et al., 2009).

In terms of the intervention forms, there were three main categories: group intervention, individual intervention, and mixed intervention. Fourteen studies were conducted using group interaction interventions (e.g., Harris and Bodden, 1978; Constantino, 1988; Lökk, 1990; Ollonqvist et al., 2008; Routasalo et al., 2009), seven studies used individual interventions (e.g., Schulz, 1976; Heller et al., 1991; Brennan et al., 1995; MacIntyre et al., 1999; Yi et al., 2012), and five studies combined the above two approaches (Drentea et al., 2006; Hang et al., 2011). The three intervention types could be subclassified into seven subtypes: intervention activities provided, group intervention training support, group intervention in remote service, face-to-face individual intervention, individual interventions in remote service, mixed interventions in remote service, and mixed interview intervention. Among them, seven items were group intervention activities-provided studies (e.g., Harris and Bodden, 1978; Constantino, 1988; Lökk, 1990; Ollonqvist et al., 2008; Routasalo et al., 2009), eight items were group intervention training support studies (Fukui et al., 2003; Savelkoul and de Witte, 2004; Kremers et al., 2006; Bøen et al., 2012; Saito et al., 2012), one item was a group intervention in remote service study (White et al., 2002), three items were face-to-face individual intervention studies (Schulz, 1976; MacIntyre et al., 1999; Yi et al., 2012), four items were individual interventions in remote service studies, two items were mixed interventions in remote service studies (Mountain et al., 2014; Czaja et al., 2017), two items were mixed interventions in remote service studies (Drentea et al., 2006; Hang et al., 2011), and three items were mixed interview intervention studies.

With regard to the time and frequency of intervention, most of the intervention frequency was regular, one time or two times per week. Most interventions lasted from 6 weeks to 1 year, and there was one study that lasted 5 years (Drentea et al., 2006); one study did not elaborate on the intervention frequency information (Heller et al., 1991). Among them, the primary recipient of the intervention included caregivers, disabled people, family members, older adults, and older adults living alone in the community. Only 50% (13/26) of the studies were specifically focused on social isolation or isolation (e.g., Harris and Bodden, 1978; Savelkoul and de Witte, 2004; Routasalo et al., 2009; Black et al., 2014; Chan et al., 2017), while the rest of the studies were secondary or indirect observations of variables. Intervention practitioners were health commissioners or professional social workers (e.g., Lökk, 1990; Savelkoul and de Witte, 2004; Ollonqvist et al., 2008; Routasalo et al., 2009; Saito et al., 2012), teachers (White et al., 2002; Czaja et al., 2017), students (Schulz, 1976; Constantino, 1988; MacIntyre et al., 1999), or experts. One study involved all of the above staff (Bøen et al., 2012), and one study did not specify the identity of the intervention practitioner (Harris and Bodden, 1978).

In studies that featured control conditions, the control or comparison intervention included setting the control group (e.g., Constantino, 1988; Kremers et al., 2006; Ollonqvist et al., 2008; Routasalo et al., 2009; Black et al., 2014), conventional care, and waiting-list control; four studies used a variety of cross interventions (Schulz, 1976; Savelkoul and de Witte, 2004; Slegers et al., 2008; Mountain et al., 2014); and six studies conducted remote interventions (e.g., Heller et al., 1991; Brennan et al., 1995; Morrow-Howel et al., 1998; Slegers et al., 2008; Mountain et al., 2014). In addition, between 6 months and 3 years after the baseline review, seven studies conducted only one follow-up (e.g., Schulz, 1976; Harris and Bodden, 1978; MacIntyre et al., 1999; Ollonqvist et al., 2008; Black et al., 2014). Thirteen studies included two to four follow-up visits in 2 years after the intervention (e.g., Constantino, 1988; Lökk, 1990; Routasalo et al., 2009; Chan et al., 2017). One of the studies collected follow-up data 11 times during the 5 years of the study (Drentea et al., 2006).

### Methodological Quality

In order to evaluate study quality and risk of bias, the Cochrane risk-of-bias tool was applied (see Table 2). Seven studies were classified as low risk of bias (e.g., Savelkoul and de Witte, 2004; Ollonqvist et al., 2008; Routasalo et al., 2009; Bøen et al., 2012; Chan et al., 2017), two studies were classified as high risk of bias (Schulz, 1976; Hang et al., 2011), and the rest of the 17 studies were rated as moderate risk of bias. Two studies with high-bias risk will not be discussed further. The remaining 24 studies will be discussed in the following.

**Table 2:** Quality of RCT studies included in the systematic review.

**Authors**	**Random allocation sequence**	**Allocation concealment**	**Blinding**	**Completeness of outcome data**	**Selective reporting**	**Other sources of bias**	**Scoring**
Harris and Bodden (1978)	Unclear	Unclear	Unclear	Unclear	Yes	Unclear	7
Constantino (1988)	Yes	No	Unclear	Unclear	Yes	Yes	8
Lökk (1990)	Unclear	Unclear	Unclear	Unclear	Yes	Yes	8
Ollonqvist et al. (2008)	Yes	Yes	Yes	Unclear	Yes	Yes	11
Routasalo et al. (2009)	Yes	Yes	Unclear	Unclear	Yes	Yes	10
Black et al. (2014)	Yes	Unclear	Unclear	Yes	Yes	No	8
Chan et al. (2017)	Yes	Unclear	Yes	Yes	Yes	Unclear	10
Ristolainen et al. (2020)	Yes	Unclear	Unclear	Unclear	Yes	Unclear	8
Fukui et al. (2003)	Unclear	Unclear	Unclear	Yes	Yes	Yes	9
Savelkoul and de Witte (2004)	Unclear	Yes	Yes	Yes	Yes	Yes	11
Kremers et al. (2006)	Unclear	Unclear	Unclear	Unclear	Yes	Yes	8
Bøen et al. (2012)	Unclear	Yes	Unclear	Yes	Yes	Yes	10
Saito et al. (2012)	Unclear	Unclear	No	Yes	Yes	Yes	8
White et al. (2002)	Unclear	Unclear	Unclear	Yes	Yes	Unclear	8
Schulz (1976)	Unclear	Unclear	Unclear	Unclear	No	No	4
MacIntyre et al. (1999)	Unclear	Unclear	Unclear	Yes	Yes	Unclear	8
Yi et al. (2012)	Yes	Unclear	Unclear	Unclear	No	No	7
Heller et al. (1991)	Unclear	Unclear	Unclear	Unclear	Yes	Yes	7
Brennan et al. (1995)	Unclear	Unclear	No	Yes	Yes	Unclear	7
Morrow et al. (1998)	Unclear	Unclear	Unclear	Yes	No	Unclear	6
Slegers et al. (2008)	No	Unclear	Unclear	Yes	Yes	No	6
Mountain et al. (2014)	Yes	Unclear	No	Yes	Yes	Yes	9
Czaja et al. (2017)	Yes	Yes	Unclear	Unclear	Yes	No	8
Drentea et al. (2006)	Unclear	Unclear	Unclear	Unclear	Yes	Yes	8
Hang et al. (2011)	Unclear	Unclear	No	Yes	No	No	4
Lai et al. (2020)	Yes	Unclear	Unclear	Yes	Yes	Unclear	9

The overall quality of the study continued to improve over time. Seven out of the eight intervention studies before 2000 (e.g., Schulz, 1976; Harris and Bodden, 1978; Constantino, 1988; Lökk, 1990; MacIntyre et al., 1999) were rated as moderate risks. Since 2000, 10 of the 18 studies were moderate bias risk; among which, seven were low bias risk.

### Intervention Characteristics and Effects

Overall, outcomes labeled with “Y” means the intervention had significant effect on this variable, while “N” indicates no significant effect. Nineteen of the 24 intervention studies showed improvement in social isolation in at least one dimension (e.g., Harris and Bodden, 1978; Constantino, 1988; Lökk, 1990; Routasalo et al., 2009; Black et al., 2014). There was a diversity of definitions and methods of measuring social isolation where it was unclear on how best to categorize all outcomes that were grouped as “social isolation.” Where there was sufficient information about type of a social isolation outcome being measured, studies were categorized as structural social support (such as emotional or psychological support) or functional social support (such as instrumental support) (Table 3).

**Table 3:** Study results for outcomes of loneliness, structural social support, and functional social support.

**Author, time (country)**	**Intervention methods**	**Bias risk**	**Loneliness**	**Structural social support**	**Functional social support**	**Remarks**
Harris and Bodden (1978) (USA)	Group intervention activities	Medium	–	Y 6 weeks social contact d = 12	–	Social isolation improved within 6 weeks
Constantino (1988) (USA)	Group intervention activities	Medium	–	Y RSAS 6 weeks; 12 months d = −0.65; d = −0.27	–	In 12 months, social isolation improved, especially at week 6; all time periods, the intervention group was superior to the controlled group
Lökk (1990) (Switzerland)	Group intervention activities	Medium	*N*	Y Social network index 6 weeks; 12 weeks d = 0.8; d = 1.3	Y Availability of social contacts 24 weeks d = 6.6	At sixth weeks, social networks widened; at twelfth weeks, the effect disappeared; at twenty-fourth weeks, close friends increased
Ollonqvist et al. (2008) (Finland)	Group intervention activities	Low	N	N	–	Within 12 months, participants in the intervention group were less likely to suffer from loneliness
Routasalo et al. (2009) (Finland)	Group intervention activities	Low	N	Y found new friends 45%	–	Within 12 months, the number of friends increased
Black et al. (2014) (USA)	Group intervention activities	Medium	Y	Y	–	TCC has the capacity to alter stress levels in lonely older adults and to attenuate the rate of increase in a key transcription factor
Chan et al. (2017) (China)	Group intervention activities	Low	Y loneliness 6 months d = −1.84	Y social support 6 months d = 5.4	Y social network 6 months not clear	Reported a significantly greater improvement on the loneliness scale
Ristolainen et al. (2020) (Finland)	Group intervention activities	Low	Y UCLA(loneliness) 6 months d = −0.1	Y social relationship 6 months d = 2.5	–	Within 6 months, loneliness were improved but social contect were not improved
Fukui et al. (2003) (Japan)	Group intervention training support	low	Y UCLA(loneliness) 6 months d = −2.9	Y the number of confidants 6 months d = 1.8	Y the satisfaction with confidants 6 months d = 0.4	Within 6 months, loneliness was reduced, self-confidence increased, and mutual satisfaction improved
Savelkoul and de Witte (2004) (Norway)	Group intervention training support	Low	N	N	–	Within 6 months, only social skills increased, while loneliness, social networks, and well-being were not improved
Kremers et al. (2006) (Norway)	Group intervention training support	low	Y social loneliness 6 weeks d = −2.0	N	–	Within 6 months, overall efficacy and emotional isolation were not improved; Social isolation improved for sixth weeks, and disappeared within sixth months.
Bøen et al. (2012) (Norway)	Group intervention training support	Low	–	Y made new friends 40%	Y feeling of social support 12 months d = 0.65	Within 12 months, social support improved significantly, depression increased, life satisfaction decreased, the intervention group was better than the control group, and there was no change in health status
Saito et al. (2012) (Japan)	Group intervention training support	Low	Y AOK loneliness scale 6 months d = −1.0	Y social support 6 months d = 0.6	-	6 months, increased social support, social contact, and social activities to improve is not obvious, increased awareness of community service, increased loneliness, depression has not changed.
White et al. (2002) (USA)	Groups intervene in remote services	Low	N	N	–	Within 5 months, loneliness was not improved, and the number of intimate friends remained unchanged
MacIntyre et al. (1999) (Canada)	Individual intervention interview	Low	–	Y social integration 6 weeks d = 3.08	N	Within six weeks, social inclusion has enhanced, there is no improvement in intimacy and so on.
Yi et al. (2012) (China)	Individual intervention interview	low	Y UCLA 6months d = −8.09	–	Y family ties increased not clear	Within 6 months, loneliness and depression were significantly improved, and family ties increased
Heller et al. (1991) (United States)	Individual intervention in remote service	Low	N	–	N	Loneliness was not improved in 20 or 30 weeks, and friends and relatives showed no improvement
Brennan et al. (1995) (USA)	Individual intervention in remote service	Low	N	–	–	12 months, the social loneliness has no effect
Morrow et al. (1998) (USA)	Individual intervention in remote service	Low	N	Y person contact 4 months not clear	N	Within 4 months, social contacts increased, but social satisfaction was not improved, and unmet need declined within 8 months
Slegers et al. (2008) (Norway)	Individual intervention in remote service	low	N	N	–	Within 12 months, there was no improvement in loneliness or social network across all 3 control groups
Mountain et al. (2014) (UK)	Mixed intervention in remote service	Low	N	Y social function 6 months d = 13.4	–	Within 6 months, there was no improvement in loneliness, but the social function was improved
Czaja et al. (2017) (USA)	Mixed intervention in remote service	Low	Y loneliness 12 months d = −2.5	Y social support 6 months 1.33	–	Access to technology applications such as PRISM may enhance social connectivity and reduce loneliness among older adults
Drentea et al. (2006) (United States)	Mixed intervention	Low	–	–	Y satisfaction with social support not clear	Within 5 years, social support satisfaction improved
Lai et al. (2020) (Canda)	Mixed intervention	medium	Y loneliness 10 weeks d = −0.63	Y barriers to social participation 10 weeks d = 0.43	–	Within 10 weeks loneliness and barriers to social participation were improved

–*, no report; Y had statistical significance; N, no statistical significance*.

### Intervention Effects According to Intervention Methods

Generally, according to the classification of different intervention methods, there are 14 group interventions, 8 of them were group activities, 5 of them focus on social support training, and 1 was conducted in a remote manner. Moreover, there are six individual interventions; two of them are interviews, and the rest of four are remote service. In addition, there are four mixed interventions, and two of them are remote service.

As to intervention methods, six of the eight group intervention activities improved structural social support (Harris and Bodden, 1978; Constantino, 1988; Lökk, 1990; Routasalo et al., 2009; Black et al., 2014; Lai et al., 2020), while various forms of outcome measures were conducted. For instance, one low-risk physical exercise study showed no obvious improvement in loneliness and structural social support (Ollonqvist et al., 2008), while Taiji physical exercise showed great effect (Black et al., 2014; Chan et al., 2017). Three group interventions focused on functional social support reported significant improvements (Fukui et al., 2003; Bøen et al., 2012; Saito et al., 2012). Four structural social support studies (Fukui et al., 2003; Savelkoul and de Witte, 2004; Kremers et al., 2006; Saito et al., 2012) reported that two out of four patients had no improvement effect or the effect disappeared over time, while a few studies reported significant effects (Fukui et al., 2003; Saito et al., 2012). A moderate risk bias group intervention conducted earlier with remote services found no improvement (White et al., 2002), while another mixed remote service intervention had effects on structural social support (Mountain et al., 2014). Two individual studies that involved face-to-face interviews showed significant improvement in structural social support (MacIntyre et al., 1999; Yi et al., 2012). One of the four older (before 2010) individuals involved in a remote service study (Heller et al., 1991; Brennan et al., 1995; Morrow-Howel et al., 1998; Slegers et al., 2008) showed improvement effects on structural social support, but the follow-up effect was very short (Morrow-Howel et al., 1998). Two studies with moderate risk bias conducted by mixed intervention showed improvement in functional social support (Czaja et al., 2017; Lai et al., 2020).

### Intervention Effects According to Intervention Environment

The external environment of intervention, such as the intervention practitioner, the place of intervention, and the client, was also an important factor in the effect of intervention. Five of the six interventions provided by experts showed improved outcomes (Heller et al., 1991; Fukui et al., 2003; Kremers et al., 2006; Mountain et al., 2014; Czaja et al., 2017). Seven of the 10 interventions provided by health or social workers were also effective (Lökk, 1990; Ollonqvist et al., 2008; Saito et al., 2012; Black et al., 2014; Chan et al., 2017; Lai et al., 2020; Ristolainen et al., 2020). Four interventions provided by teachers or students of the education community presented improved results (Constantino, 1988; MacIntyre et al., 1999; White et al., 2002; Slegers et al., 2008). In addition, one study multiple types of intervention practitioners (Bøen et al., 2012), another study didn't specifically described the information of intervention providers (Harris and Bodden, 1978).

As for the field of intervention, five studies took place in schools or public places (Harris and Bodden, 1978; Constantino, 1988; Routasalo et al., 2009; Black et al., 2014; Chan et al., 2017), and 1~2 dimensions were improved. Five studies were professional treatment interventions (e.g., senior center, rehabilitation center, and hospital) (Lökk, 1990; Ollonqvist et al., 2008; Routasalo et al., 2009; Bøen et al., 2012; Black et al., 2014), and all of these showed improvements in 1~3 dimensions. In six individual studies of private residences (Heller et al., 1991; Brennan et al., 1995; Morrow-Howel et al., 1998; MacIntyre et al., 1999; Yi et al., 2012; Lai et al., 2020), only two intervention studies presented no improved effects, even any dimension (Heller et al., 1991, Brennan et al., 1995). The other three studies did not provide evidence of the implementation environment (Savelkoul and de Witte, 2004; Kremers et al., 2006; Ristolainen et al., 2020). In addition, studies precisely targeting clients with social isolation or loneliness problems had better effects on all dimensions (e.g., Harris and Bodden, 1978; Savelkoul and de Witte, 2004; Routasalo et al., 2009; Bøen et al., 2012; Saito et al., 2012). By contrast, the studies without specific targets showed a worsened effect (Savelkoul and de Witte, 2004).

### Intervention Effects According to Duration of Effect

Among the 14 studies reporting structural social support effect, three of them used social support as the outcome (Saito et al., 2012; Chan et al., 2017; Czaja et al., 2017), two studies observed the change of new friend number as an outcome (Routasalo et al., 2009; Bøen et al., 2012). One study showed that 45% of the participants made new friends in 1 year (Routasalo et al., 2009), while another showed that 40% of the participants made new friends in 1 year (Bøen et al., 2012), two studies reported using social contact as an outcome (Harris and Bodden, 1978; Morrow et al., 1998). And all studies reporting functional social support effect took completely different indicators during 6–12 months.

## Discussion

This study found substantial heterogeneity in the interventions delivered, and the overall quality of included studies indicated a low to medium risk of bias. Also, group intervention activities and individual intervention were effective in improving structural social support; mixed intervention and group intervention on training support significantly improved functional social support. We found that the interventions with accurate targeting of clients in social and public places had more obvious effect. Interventions in which older people were active participants also appeared more likely to be effective. In addition, professionals were better than teachers and students in conducting intervention. The findings provide a tentative indication of the potential benefits of specific types of intervention for improving loneliness/social isolation, advancing theory-informed development of interventions and improving design of evaluation studies. The remote service interventions were debatable, as the recent studies have showed improvement in structural social support, but no effect on older studies. Because of the contradictory results, more research is needed to examine the complexity of “remote interventions” from the perspective of process evaluation. Interventions conducted in social and public places had better effects, and interventions with accurate targeting of clients had more obvious effects. Studies evaluating interventions delivered by professional practitioners appeared to yield better outcomes than those where the intervention was delivered by non-professionals. Effective intervention for older adults in isolation not only improved structural social support, functional social support, and mitigation of loneliness but also promoted the health of older adults.

In the experimental studies, there were a variety of interventions on social isolation. Although experimental design is not always feasible or accepted by participants, this kind of study can provide a scientific and normative reference for the implementation process and assessment report, promote the utilization of randomized control trials, improve the design level, standardize the research process, improve the quality of evidence, and provide a reference for policy-making. We advocate professionals to provide face-to-face intervention in the field of daily life rather than in the home environment and recommend that more efficient remote interventions within smart terminals be developed to achieve better results.

In real life, the environment preference of older adults has an obvious effect on their social interaction. Older adults who enjoy being alone are more likely to be socially isolated. The incidence of social isolation among older adults in different living conditions was also different, with those who were widowed, had low income, and in poor health, more likely to feel lonely and socially isolated. In addition, as age increases, older adults can be more dissociated from social interaction and prefer to be isolated (Lu et al., 2013). Therefore, when we design social isolation interventions, it is essential to consider personal preference, living status, and physiological characteristics of older adults and adjust measures accordingly so as to promote the effectiveness of the intervention. In addition, well-designed remote intervention system, such as personal reminder information and social management (PRISM) system, has the potential to change attitudes toward technology and increase technology self-efficacy.

At the policy level, the establishment of social support systems is imminent (Liu and Ni, 2002). With the advancement of family planning policy, such as China, the aging of the population is becoming more and more serious, and the “4-2-1” or “4-2-2” family pattern (4-grandparent, 2-parent, and 2- or 1-child) has gradually formed (Nan and Dong, 2019). Family support functions have greatly weakened, and, especially, the needs for social interaction and spiritual comfort are not satisfied. Therefore, we must establish a community-based pension support service platform, develop professional social work vigorously, cooperate with research institutes to obtain scientific evidence in order to address the problem of social isolation of older adults, improve their physical and mental health, as well as quality of life, and promote the healthy aging of the population.

## Limitations and Future Research Directions

In this study, the inclusion literature was defined as older adults over age 50, who have been in isolation or loneliness. However, the relevant research on the concept of social isolation does not use a standardized and unified definition, so inclusion bias may have been incorporated. Although the inclusion criteria were designed to reduce social isolation or loneliness, only 14/24 studies specifically addressed the problem (e.g., Constantino, 1988; Li and Zhou, 2002; Savelkoul and de Witte, 2004; Kremers et al., 2006; Chan et al., 2017). The study may also have the potential risk that the assessment of social isolation or loneliness was due to other characteristics of the target client (Liu and Ni, 2002). Restricting the study language to English and Chinese may have increased the inclusion bias. The quality and expression of the research in the historical period also limited the quality of this study. Some studies conducted a qualitative report rather than quantitative data. It is not appropriate to use quantitative methods as well as meta-analysis due to the heterogeneity of the study subjects.

At the same time, we found that most pieces of randomized controlled trial research in this field were from developed countries. Future research not only needs to enrich the original evidence from all over the world but especially from developing countries. In addition, most of the pieces of research from Finland, Norway, the United States, and other developed countries were different from developing countries due to legal or volunteer service organizations; thus, the applicability and the effectiveness of the evidence are worth discussing further. Moreover, more refined subgroups of systematic review can be done in the near future; for example, systematic review could be used to quantify the effect of intervention on a certain type of intervention.

## Conclusion

The findings provide a tentative indication of the potential benefits of specific types of intervention for improving loneliness/social isolation, advancing theory-informed development of interventions, and improving design of evaluation studies.

Firstly, this study suggests that group and mixed intervention targeting of older adults could be helpful for alleviating social isolation problems. The use of modern technology for remote services could also present good results. Moreover, our systematic review has identified a need for well-conducted studies to improve the evidence base regarding the effectiveness of social interventions for alleviating social isolation. However, more well-conducted RCTs of the effectiveness of social interventions for alleviating social isolation are needed to improve the evidence base.

Because of the debating results, further research is needed to examine the effect of remote interventions from the perspective of process evaluation.

## Data Availability Statement

The original contributions presented in the study are included in the article/supplementary material, further inquiries can be directed to the corresponding author/s.

## Author Contributions

FT is responsible for analyzing data and writing the draft of the results. CLY is responsible for analyzing the data. FF is responsible for revising the manuscript and submitting the paper. LSW is responsible for writing the literature review. IC is responsible for providing suggestions for revising the paper. All authors contributed to the article and approved the submitted version.

## Funding

2019 National Social Science Fund (19BSH173): The Evidence Reorganization, Conversion, and Enlightenment Research of Evidence-based Social Work Intervention on Long-term Care of older adult.

## Conflict of Interest

The authors declare that the research was conducted in the absence of any commercial or financial relationships that could be construed as a potential conflict of interest.

## Publisher's Note

All claims expressed in this article are solely those of the authors and do not necessarily represent those of their affiliated organizations, or those of the publisher, the editors and the reviewers. Any product that may be evaluated in this article, or claim that may be made by its manufacturer, is not guaranteed or endorsed by the publisher.
